# The Prediction of the Concentration of Artificially Added Microplastics in Different Foods and Feed Ingredients Using Near-Infrared Spectroscopy—Instrument Type, Limit of Detection and Quantification

**DOI:** 10.3390/foods15213857

**Published:** 2026-10-28

**Authors:** Daniel Cozzolino, Adam Kolobaric, Shanmugam Alagappan, Joseph Robert Nastasi, Louwrens C. Hoffman

**Affiliations:** 1 Centre for Nutrition and Food Sciences, Queensland Alliance for Agriculture and Food Innovation (QAAFI), The University of Queensland, Brisbane, QLD 4072, Australialouwrens.hoffman@uq.edu.au (L.C.H.); 2 School of Agriculture and Food Sustainability (AGFS), The University of Queensland, Brisbane, QLD 4072, Australia

**Keywords:** calibration, microplastic, feed, food, validation, LOD, LOQ

## Abstract

Predicting microplastic (MP) concentrations in different foods and feed ingredients is a growing area of interest in near-infrared (NIR) spectroscopy. The objective of this paper is to evaluate the ability of NIR spectroscopy (benchtop and portable instruments) to predict the concentration (0% to 5% *w*/*w*) of the addition of polymers [polyethylene (PE), polypropylene (PP), and polyethylene terephthalate (PET)] into three different matrices commonly encountered as food and feed ingredients. This study used samples of banana, spinach, breadcrumbs, and black soldier fly larvae (BSFL) meal with different levels of polymer addition. Calibration models to predict the concentration of MP in the samples were developed using partial least squares (PLS) regression. The coefficient of determination in cross-validation (R^2^_CV_) and the standard error in cross-validation (SECV) were 0.76 and 0.72%, and 0.81 and 0.75% using cross-validation (CV) and the test set, respectively. Using CV and the test set, the R^2^_CV_ and SECV were 0.92 (SECV: 0.38%) and 0.95 (SECV: 0.32%), respectively, for the portable instrument. The R^2^_CV_ and SECV were 0.81 (SECV: 0.59%) and 0.81 (SECV: 0.60%) for the CV and test sets analysed using the benchtop instrument, respectively. The SEP was 0.41% and 0.65% using the portable and benchtop instruments, respectively. These results demonstrate the potential of both portable and benchtop NIR spectroscopy as rapid tools for predicting microplastic concentrations in a range of food and feed matrices.

## 1. Introduction

Vibrational spectroscopy [e.g., mid (MIR) and near (NIR) infrared, Raman spectroscopy] has been used as a tool to identify different polymers in biological (e.g., food ingredients and products) [1,2] and environmental samples (e.g., soils) [3,4,5]. In these applications, the plastic or polymer is usually extracted from the sample matrix and cleaned, after which the type of polymer is then identified using instruments such as Raman and MIR infrared spectroscopy as well as hyperspectral or multispectral imaging systems [3,4,5,6]. The main objective of these applications is to overcome the so-called human errors that occur during the visual recognition of microplastics. However, microplastic analysis also commonly relies on sample-preparation and separation procedures, such as flotation and density separation, which can introduce additional sources of variability and contribute to differences in the results obtained among analytical workflows [3,4,5,6]. Furthermore, these applications do not fully address or explore the ability of vibrational spectroscopy (e.g., molecular and bond information about the samples) to develop either untargeted or targeted models to classify and quantify microplastics (MP) and polymers in a wide range of samples.

Recent years have shown an increased interest in the field of NIR spectroscopy for the prediction of the concentration of MP or related polymers in different food products, feed ingredients and other types of samples (e.g., soil, water) [7,8,9]. In this context, several scientific papers have reported the ability of NIR to predict the presence and concentration of polymers in soil samples, fish and seafood, and a wide range of food ingredient and product samples [7,8,9,10]. However, most of these have evaluated microplastic or polymer prediction within a single sample matrix, leaving the ability of NIR spectroscopy to develop robust predictive models across multiple, compositionally distinct food and feed matrices largely unexplored.

Food safety and security are important issues facing the modern food industry [11,12]. Detecting and quantifying MP and polymers in food ingredients and products is therefore important for the industry, as they affect the safety of food ingredients and products for consumers [11,12]. Furthermore, methods and techniques that are easy to use across the value and supply chains are also important to ensure the safety of food products and ingredients [11,12]. Several authors and practical applications have demonstrated the ability of NIR to analyse a wide range of food ingredients and products to ensure their composition (e.g., protein content), safety and integrity [11,12]. The availability of handheld and portable instruments has also supported developments of NIR spectroscopy moving away from the laboratory setting and into the field [11,12,13]. Despite the increasing application of NIR spectroscopy and the growing body of research demonstrating its potential, several methodological factors that can influence model performance remain insufficiently considered and evaluated. Some of these issues include the use of cross-validation or validation using independent datasets, the type of sample analysed and the sample presentation method (e.g., dry or high moisture samples), and instrumentation limitations (e.g., optical geometry, instrument characteristics, and wavelength range) [14,15,16].

This study evaluated the ability of benchtop and portable NIR instruments to predict added polymer concentrations across three compositionally distinct food and feed matrices (e.g., banana-spinach mixtures, breadcrumbs, and black soldier fly larvae (BSFL) meal). Emphasis was placed on determining whether robust calibration models could be developed across multiple matrices rather than within a single sample type, and on comparing the predictive performance of portable and benchtop NIR instruments.

## 2. Materials and Methods

Samples were obtained from three different experiments and combined into two different datasets (e.g., polymer addition, different instrument concentrations). Details about the samples, the concentration of microplastics added to the samples (from 0% up to 5% *w*/*w*), and the type of microplastic [polyethylene (PE), polyethylene terephthalate (PET) and polypropylene (PP)] used were reported elsewhere [17,18]. Polymer pellets (PE, PET, and PP; Scientific Polymer Products, Inc., Ontario, NY, USA) were cryogenically frozen (−80 °C) to induce embrittlement and milled using a Tissuelyser II (Qiagen, Tokyo, Japan) at 30 Hz for 5 min to produce particles <1 mm. Mixtures were prepared by adding the polymer at different concentrations to the food or feed ingredient, from 0.1 to 5.0% w/w, depending on the mixture. These ranges simulated typical contamination levels reported in food matrices (0.1–1.0%) for monitoring purposes, as well as less frequent but more severe contamination events (1.5–5.0%) [17,18]. For example, a 5% *w*/*w* mixture was created by weighing 0.05 g of the polymer and 0.95 g of the food/feed and mixing thoroughly. The spectra of the samples were collected using a benchtop and a portable spectrometer in reflectance mode. The benchtop instrument was an FT-NIR Tango equipped with a gold-coated integrating sphere (Tango; Bruker Optics GmbH, Ettlingen, Germany), where sample spectra were recorded in reflectance using an integration time of 64 interferogram scans across 11,550–3950 cm^−1^ with a spectral resolution of 4 cm^−1^. The instrument diagnostics and scanning were performed using OPUS software (version 8.5, Bruker Optics GmbH, Ettlingen, Germany). The portable instrument was a MicroNIR portable instrument (Viavi Solutions Inc., Chandler, AZ, USA) operating in the wavelength range 900 to 1600 nm, with an integration time of 50 ms and an average of 50 scans per spectrum.

The NIR spectra were exported from different commercial proprietary formats into Vektor Direktor (version 2.0, KAX Group, Sydney, NSW, Australia) for data analysis. The NIR spectra of the samples were processed using Savitzky–Golay (second derivative, eleven-point window, second-order polynomial) [19] before analysis. Partial least squares regression (PLS) was developed using cross-validation (leave-one-out) [20,21]. Two datasets were used, one combining banana, breadcrumbs, black soldier fly larvae meal, and spinach (controls and with different levels of MP) and scanned on the benchtop instrument. A second set included only banana, breadcrumbs, and spinach samples (controls and with different levels of MP), was analysed and was used to compare both benchtop and portable instruments. The dataset (*n* = 500) was split into calibration (CAL) and validation (VAL) sets using the Kennard-Stone (KS) algorithm, resulting in calibration (*n* = 250) and validation (*n* = 250) sets. The CAL models were developed using cross-validation (CV) and evaluated using the coefficient of determination in cross-validation (R^2^_CV_), the standard error in cross-validation (SECV), the standard error of prediction (SEP), bias and slope [20,21]. In addition, the models’ ability to predict the concentration of MPs was evaluated using the residual predictive deviation (RPD) values, calculated as the ratio of standard deviation (SD) to the SECV or SEP [20,21]. The RPD value is used as an indicator of the predictive performance of the model, where values greater than three are generally considered adequate in quality control applications of NIR spectroscopy [20,21].

The limit of detection (LOD) and limit of quantification (LOQ) were calculated using the following equations [22,23,24], where the RMSEP is defined as the root mean square of the standard error in prediction. The RMSEP measures the total error in the model, both the random error and systematic bias (RMSEP^2^ = SEP^2^ + bias^2^) [25].

LOD = 3.3 × RMSEP

LOQ = 10 × RMSEP

## 3. Results and Discussion

**Figure 1:**
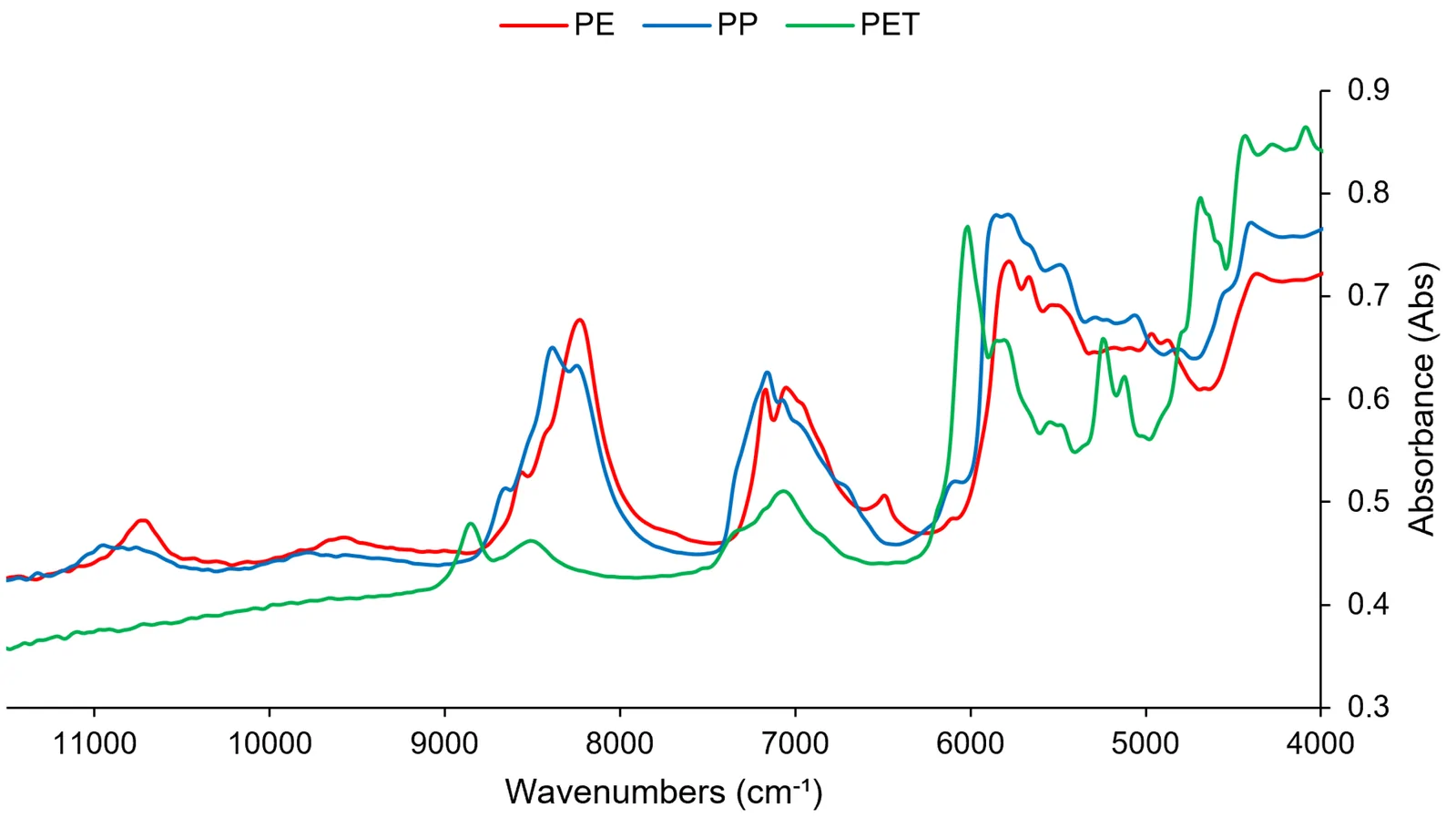
Near-infrared (NIR) absorbance spectra of pure polyethylene (PE), polyethylene terephthalate (PET) and polypropylene (PP) acquired using a benchtop NIR instrument.

The NIR absorbance spectra of pure PE, PET and PP particles (<1 mm), acquired using the benchtop NIR instrument, are presented in Figure 1. The spectra contained distinct absorption features indicative of differences in the molecular structures of the three polymers. PE and PP showed relatively similar spectral profiles, with prominent absorption bands at approximately 8200–8400, 6900–7300 and 5700–6150 cm^−1^, predominantly associated with overtone and combination vibrations of C–H bonds [26]. The bands at approximately 5700–6150 and 8200–8400 cm^−1^ correspond to the first and second overtones of C–H stretching, respectively, whereas the region at 6900–7300 cm^−1^ is associated with combination vibrations involving C–H stretching and deformation [17,18,26]. In contrast, PET exhibited a distinct spectral profile, particularly in the regions around 5900 and 4500–4700 cm^−1^, reflecting differences in its aromatic and ester-containing molecular structure. Only spectra acquired using the benchtop instrument are presented, as its broader spectral range and higher spectral resolution provided a better representation of the characteristic absorption features of the polymers compared to portable NIR spectra.

**Figure 2:**
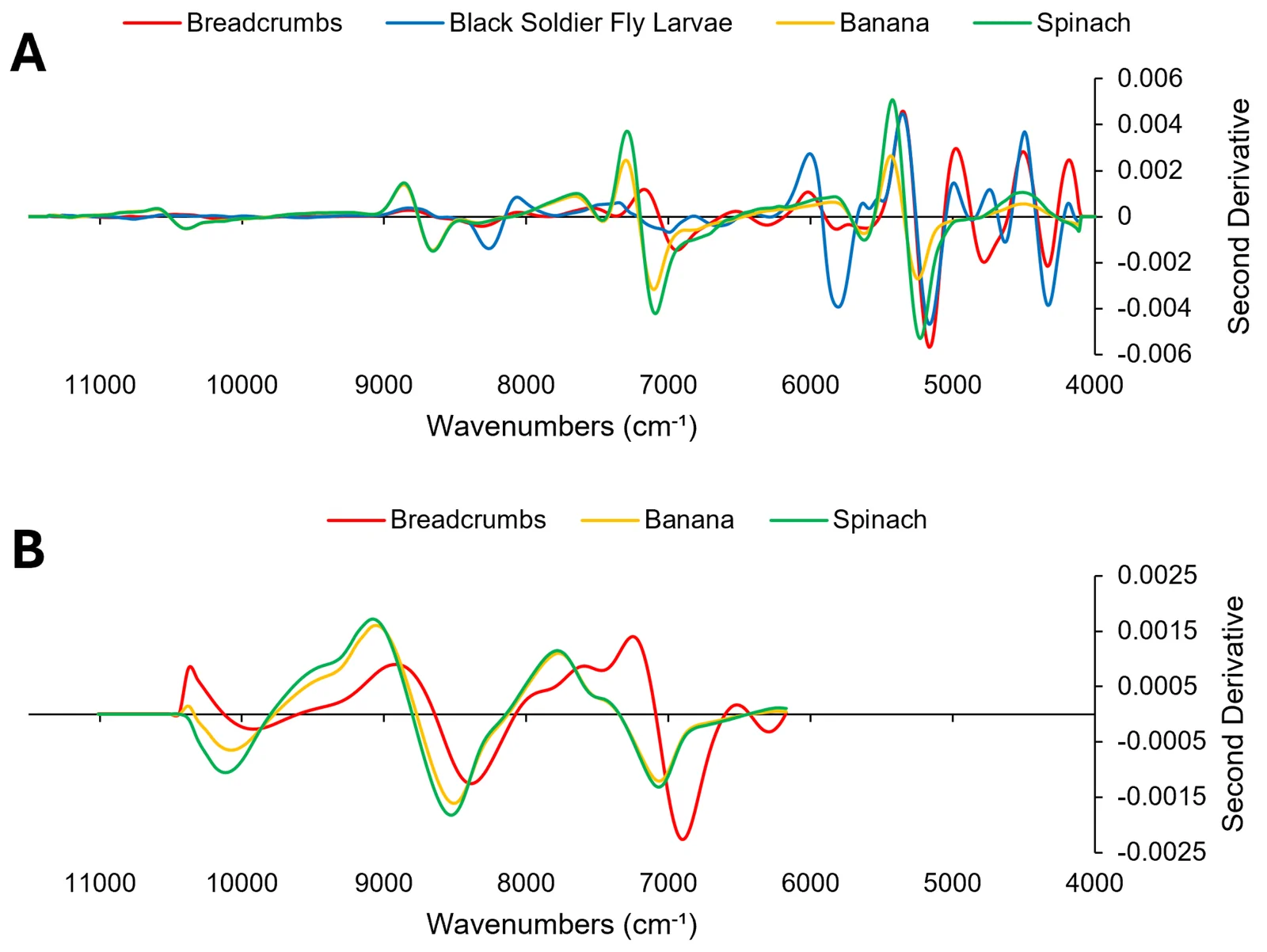
Second derivative near-infrared spectra of the samples analysed using the benchtop (**A**) and portable (**B**) instruments.

Figure 2 reports the second derivative NIR spectra of the mixture (e.g., polymer + banana, breadcrumbs, BSFL meal, or spinach) samples collected using either the portable or benchtop instruments. The NIR second derivative spectra of the samples analysed using the benchtop instrument (Figure 2A) showed differences at wavenumbers around 8240 cm^−1^ (CH_2_), around 6946 cm^−1^ (CH and CH_2_, and O-H aromatic groups), at 5824 cm^−1^ (CH_2_), and at 5184 cm^−1^ (O-H from large molecules such as polysaccharides). Around 4820 cm^−1^ (COON_2_ associated with peptide groups, N-H and C=O groups associated with compounds having nitrogen), and around 4368 cm^−1^ (CONH_2_ associated with peptide groups, and C=O groups associated with peptides) [17,18,26]. The second derivative of the samples analysed using the portable instrument (Figure 2B) showed main absorption bands at wavenumbers around 8340 cm^−1^, 8290 cm^−1^, 7855 cm^−1^, and 7352 cm^−1^, associated with CH_3_ methyl groups, CH methyl groups, and CH groups, respectively [17,18,26,27,28]. These wavenumbers can be associated with polymers and bread’s chemical composition [26,27,28,29,30]. Furthermore, the wavenumbers around 6973 and 6293 cm^−1^ can be attributed to the presence of combination bands of CH vibrations, associated with CH_2_ and CH_3_ groups, and the first overtone of OH stretching, linked to both water and phenolic compounds found in bread and green vegetables (e.g., spinach) (Ar-OH), as reported [26,27,28].

Table 1 shows the cross-validation and prediction statistics for the measurement of the concentration of microplastic added in the combined set of BSFL, banana, spinach, and breadcrumb samples analysed using the NIR benchtop instrument. The cross-validation coefficient of determination (R^2^_CV_) and standard error (SECV) were 0.76 and 0.72%, and 0.81 and 0.75%, using the CV and test set, respectively. The standard error of prediction (SEP) using the validation set was 0.78%.

Table 2 summarises the calibration, cross-validation and independent validation statistics for the prediction of MP concentrations in the combined dataset comprising banana, spinach and breadcrumb samples, with BSFL meal excluded from this analysis. For the portable NIR instrument, CV yielded an R^2^_CV_ of 0.92 and an SECV of 0.38%, while CAL achieved an R^2^_CV_ of 0.95 and an SECV of 0.32%. Independent validation yielded an R^2^ of 0.91 and a SEP of 0.41%. For the benchtop instrument, the corresponding R^2^_CV_ for CV, CAL and VAL were 0.81, 0.81 and 0.78, with respective errors of 0.59%, 0.60% and 0.65%. The RPD values for independent validation were 3.6 and 2.3 for the portable and benchtop instruments, respectively. The portable instrument demonstrated superior predictive performance, achieving higher coefficients of determination and lower prediction errors.

The RPD was used to evaluate the predictive power of the calibration models, with values ranging between 2 and 4.7. The portable instrument produced the best calibration and prediction metrics (RPD > 4). Regardless of the dataset used to develop the models, the benchtop instrument produced the lowest RPD values compared with the portable instrument.

**Figure 3:**
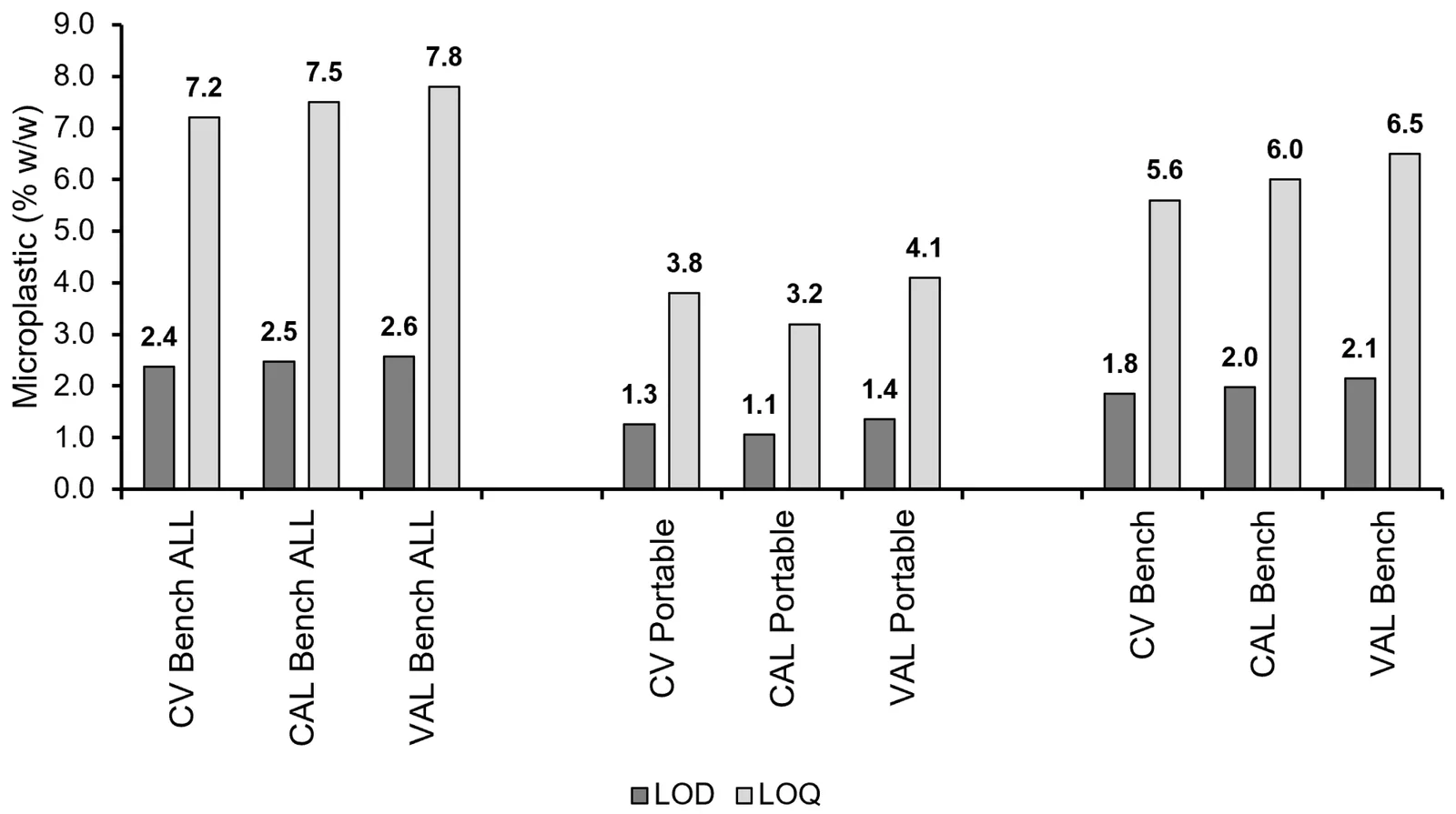
Limit of detection (LOD) and limit of quantification (LOQ) for the different dataset combinations and types of instruments used to analyse the samples. CV: Cross-validation, CAL: Calibration, and VAL: Validation.

The LOD is an important characteristic to consider when developing any analytical method. This metric is used to compare different techniques or methods and to choose the best analytical method [23,24]. The LOQ is also an important metric that indicates the lowest reliably detected concentration [23,24]. In this study, both the LOD and LOQ values were calculated using the equation presented in the Materials and Methods section. The LOD and LOQ values calculated ranged between 1.1% and 2.6%, while the LOQ value ranged between 3.2% and 7.8%. Figure 3 shows the LOD and LOQ values from the different datasets and instruments used to analyse the samples. It was observed that the portable NIR instrument yielded better LOD and LOQ values than the benchtop instrument for the datasets examined in this study. It should be noted that the reported LOD and LOQ values were estimated from the RMSEP and represent indicative model-error-derived thresholds rather than experimentally validated analytical limits. In particular, the estimated LOQ of 7.8% exceeded the maximum polymer concentration evaluated (5.0%), indicating that reliable quantification at this threshold could not be established within the investigated calibration range. Furthermore, the relatively high estimated detection and quantification limits highlight the current limitations of NIR spectroscopy for monitoring microplastic contamination at environmentally relevant concentrations. The present findings should be interpreted as a feasibility assessment of polymer concentration prediction across different food and feed matrices, with further optimisation and validation required to establish applicability at trace concentrations.

**Figure 4:**
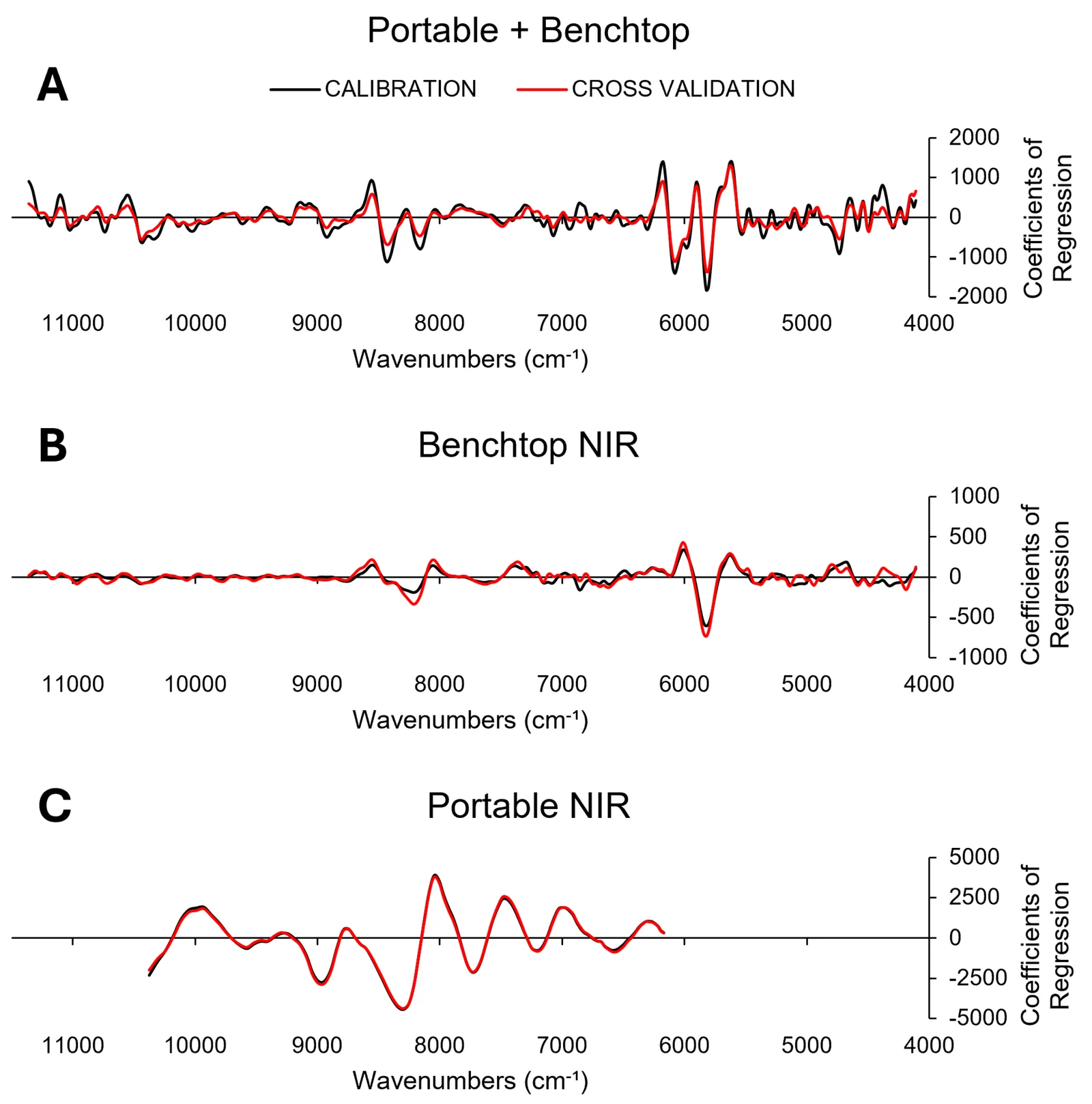
Coefficients of regression from the cross-validation and calibration models developed using independent datasets and analysed using all samples and benchtop (**A**), calibration set in benchtop (**B**) and portable (**C**) instruments.

The coefficient of regression using the benchtop instrument for all samples and the mixture of banana, spinach and breadcrumbs is shown in Figure 4A,B. The coefficient of regression using the benchtop instrument was found at wavenumbers around 8240 cm^−1^ (CH_2_), around 6946 cm^−1^ (CH and CH_2_, and O-H aromatic groups), and at 5824 cm^−1^ (CH_2_), at 5184 cm^−1^ (O-H from large molecules such as polysaccharides), at 4820 cm^−1^ (COON_2_ associated with peptide groups, N-H and C=O groups associated with compounds having nitrogen), and around 4368 cm^−1^ (CONH_2_ associated with peptide groups, and C=O groups [26,27,28,29,30,31].

The coefficients of regression obtained using the portable instrument (Figure 4C) showed main absorption bands at wavenumbers around 8992 cm^−1^, 8298 cm^−1^, 7692 cm^−1^, 7220 cm^−1^, and 6578 cm^−1^ associated with CH_3_ methyl groups, CH methyl and CH groups, respectively [26,27,28,29,30,31,32,33]. Wavenumbers observed around 6973 cm^−1^ and 6293 cm^−1^ can be attributed to the presence of combination bands of CH vibrations, associated with CH_2_ and CH_3_ groups, and the first overtone of OH stretching, linked to both water and phenolic compounds found in the samples analysed (ArOH), as reported by other authors [26,27,28,29,30,31,32,33].

It is important to note that the MPs were artificially added to different food and feed ingredient matrices under controlled laboratory conditions. The methodology followed in these experiments was considered useful for method development; however, it does not fully represent real contamination by MPs in food products and feed ingredients. The comparison between portable and benchtop NIR instruments to predict the concentration of MPs or polymers added to the samples was also evaluated using different optics/instruments. Differences observed in the calibration statistics (e.g., SECV and RPD values) might be explained by the inherent variations between instruments, such as illumination area and optical geometry, like the use of an integrating sphere (benchtop instrument) compared with the direct window reflection in the portable instrument. Furthermore, the reduced water absorption interference in the short-wave NIR region might also contribute to explaining the observed differences in the statistics (e.g., improved cross-validation in the portable instrument).

## 4. Conclusions

The present study evaluated the quantitative prediction of PE, PET and PP concentrations across compositionally distinct food and feed matrices using both benchtop and portable NIR instruments. Furthermore, the use of independent validation provides additional evidence of the predictive performance of the developed models. The results demonstrated that calibration and validation performance varied according to the composition of the dataset, validation strategy and NIR instrument used. For the combined dataset comprising BSFL meal, banana, spinach and breadcrumbs, the benchtop instrument achieved an R^2^_CV_ of 0.76 (SECV: 0.72%) and an R^2^ in validation of 0.78 (SEP: 0.78%). For the dataset excluding BSFL meal, the benchtop instrument achieved an R^2^_CV_ of 0.81 (SECV: 0.59%) and an R^2^ validation of 0.78 (SEP: 0.65%). The portable instrument demonstrated superior predictive performance for the three-matrix dataset, achieving an R^2^_CV_ of 0.92 (SECV: 0.38%) and an R^2^ in validation of 0.91 (SEP: 0.41%), with an RPD of 3.6. The estimated LOD and LOQ varied between instruments and datasets, ranging from 1.1 to 2.6% and 3.2–7.8%, respectively, with the lowest values obtained using the portable instrument. However, these model-error-derived estimates highlight the limitations of the approach for detecting microplastic contamination at trace concentrations. To further extend the applicability of this technology, future investigations should evaluate substantially lower polymer concentrations, incorporate additional food and feed matrices, and consider variables such as polymer ageing, weathering and particle size. Further external validation using independent sample batches and previously unrepresented matrices would also provide valuable insights into model robustness and generalisability. Additionally, the transferability of calibration models between different NIR instruments should be investigated, particularly considering differences in optical configuration, spectral range and signal-to-noise ratio. Overall, this study advances the application of NIR spectroscopy as a rapid, non-destructive approach for predicting microplastic concentrations in heterogeneous food and feed materials, while highlighting the further optimisation and validation required before its implementation for routine contamination monitoring is adopted in agricultural and food sectors.

## Figures and Tables

**Table 1:** Cross-validation and prediction statistics for measuring microplastic concentration in a combined set of black soldier fly larvae meal, banana, spinach, and breadcrumb samples analysed using the benchtop instrument.

		N	R^2^_CV_	SECV	Bias	Slope	LV	RPD
Benchtop	CV	450	0.76	0.72	0.003	0.77	10	2
	CAL	230	0.81	0.75	0.003	0.78	11	2
				SEP				
	VAL	243	0.78	0.78	−0.04	0.76	-	2

CV: cross-validation; CAL: calibration; VAL: independent validation; N: number of samples; R^2^_CV_: coefficient of determination in cross-validation; SECV: standard error in cross-validation; LV: latent variables; RPD: residual predictive deviation; SEP: standard error in prediction.

**Table 2:** Cross-validation and prediction statistics for the measurement of the concentration of microplastics in the combination banana, spinach and breadcrumbs samples analysed using the portable and benchtop instruments.

		N	R^2^_CV_	SECV	Bias	Slope	LV	RPD
Portable	CV	260	0.92	0.38	0.0009	0.93	7	3.9
	CAL	129	0.95	0.32	0.003	0.95	7	4.7
				SEP				
	VAL	130	0.91	0.41	0.02	0.96	-	3.6
		N	R^2^_CV_	SECV	bias	slope	LV	RPD
Benchtop	CV	260	0.81	0.59	−0.002	0.82	6	2.5
	CAL	129	0.81	0.60	−0.0009	0.84	6	2.5
				SEP				
	VAL	130	0.78	0.65	−0.11	0.73	-	2.3

CV: cross-validation; CAL: calibration; VAL: independent validation; N: number of samples; R^2^_CV_: coefficient of determination in cross-validation; SECV: standard error in cross-validation; LV: latent variables; RPD: residual predictive deviation; SEP: standard error in prediction.

## Data Availability

The datasets generated during and/or analysed during the current study are not publicly available but are available from the corresponding author on reasonable request.
